# Protease Activated Receptors and Arthritis

**DOI:** 10.3390/ijms22179352

**Published:** 2021-08-28

**Authors:** Flora Lucena, Jason J. McDougall

**Affiliations:** Departments of Pharmacology and Anesthesia, Pain Management & Perioperative Medicine, Dalhousie University, 5850 College Street, Halifax, NS B3H 4R2, Canada; flora.lucena@dal.ca

**Keywords:** arthritis, inflammation, joint damage, proteases, pain

## Abstract

The catabolic and destructive activity of serine proteases in arthritic joints is well known; however, these enzymes can also signal pain and inflammation in joints. For example, thrombin, trypsin, tryptase, and neutrophil elastase cleave the extracellular N-terminus of a family of G protein-coupled receptors and the remaining tethered ligand sequence then binds to the same receptor to initiate a series of molecular signalling processes. These protease activated receptors (PARs) pervade multiple tissues and cells throughout joints where they have the potential to regulate joint homeostasis. Overall, joint PARs contribute to pain, inflammation, and structural integrity by altering vascular reactivity, nociceptor sensitivity, and tissue remodelling. This review highlights the therapeutic potential of targeting PARs to alleviate the pain and destructive nature of elevated proteases in various arthritic conditions.

## 1. Introduction

Musculoskeletal diseases comprise the most prevalent chronic pain conditions with arthritides accounting for the majority of these disorders [1]. Although there are over 100 different types of arthritis, the most commonly studied are inflammatory rheumatoid arthritis (RA) and degenerative osteoarthritis (OA). In RA, an individual’s immune system is dysregulated and inflammatory cells begin to destroy the host joint tissues by releasing chemical mediators into the joint. Autoantibodies such as rheumatoid factor and antibodies directed towards activated citrullinated proteins are also prominent features of RA which contribute to the degenerative process and pain [2]. The RA synovium becomes hyperplastic and this pannus invades cartilage, bone, and menisci leading to altered biochemical regulation and tissue damage. OA is characterized as an inappropriate healing response to joint tissue injury whereby cartilage exhibits focal lesions, subchondral bone is dense and fissured, osteophytes form, and the menisci can become calcified [3]. Intermittent synovitis occurs in some patients and joint inflammation is heterogeneous [4]. Pain and joint stiffness are common to all types of arthritis and limit an individual’s ability to move which could put them at risk of other co-morbidities such as diabetes, obesity, or cardiovascular disease [5,6,7].

The available treatments to manage joint diseases are currently very limited and have variable efficacy between patients. Pharmacological treatments for RA include the use of non-steroidal anti-inflammatory drugs (NSAIDs), corticosteroids, methotrexate, and biologics that inhibit cytokine reactivity [8]. With respect to OA, there are no disease modifying drugs and treatments are restricted to symptom relief. Topical NSAIDs, acetaminophen, serotonin-noradrenaline reuptake inhibitors, and opioids are typically used to treat the disease [9]. All of the drug therapies have varying degrees of effectiveness and can produce undesirable side effects. The search for new drug targets that can control pain, inflammation, and function is therefore an important area for ongoing arthritis research.

The chemical mediators responsible for generating joint disease are still being defined but are known to include prostaglandins, neuropeptides, and cytokines [10]. Emerging evidence also indicates that proteolytic enzymes can also signal pain and inflammation in joints by cleaving a specific receptor family known as the protease activated receptors (PARs; Table 1). This group consists of four G-protein coupled receptors (PAR-1 to -4) that show a unique activation characteristic [11]. In the presence of a protease, a portion of the PAR extracellular N-terminus is cleaved which then exposes a new N-terminus sequence that acts as a tethered ligand which then binds to the second extracellular loop of the receptor [12]. This conformational change to the receptor leads to intracellular signalling that will vary depending on the receptor subtype, the cleaving protease, and the downstream pathway that becomes activated. In addition to receptor activation, some proteases can disarm or inactivate the PAR receptor by cleaving the N-terminus at an adjacent site [13]. More recently, a biased signalling of PARs has been extensively described, indicating the complexity of these receptors [14,15,16].

PARs have been identified on the nerve terminals of nociceptors in multiple tissues suggesting that this receptor family is involved in pain control. Co-localization and pharmacological interaction between PAR-2 and pro-algesic transient receptor potential (TRP) channels in the pancreas [17], bladder [18], and oral mucosa [19] indicate that there is functional coupling between PARs and other known pain-modulating receptors. Proteases released from immunocytes, endothelial cells, or are part of the coagulation cascade are known to cleave neuronal PARs and modulate pain neurotransmission [12]. Other studies on the skin, gut, and airways have shown that PAR activation can lead to the secondary release or pro-algesic neuropeptides such as substance P and calcitonin gene-related peptides [20,21,22]. These multiple lines of evidence assert that PARs are an attractive target for the management of miscellaneous chronic pain conditions.

This review will outline the function of PARs and give an overview of the latest studies, implicating their role in joint diseases including RA and OA.

## 2. PAR-1

### 2.1. Receptor Pharmacology

**Figure 1:**
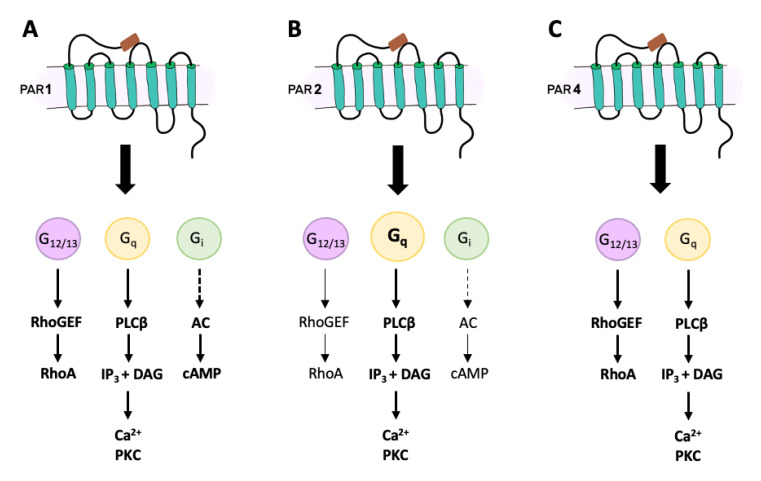
Downstream signalling pathways for PAR-1 (**A**), PAR-2 (**B**), and PAR-4 (**C**). Rho guanine nucleotide exchange factor (RhoGEF); GTPase RhoA (RhoA); phospholipase-Cβ (CPLCβ); phosphatidylinositol 4,5-bisphosphate (PIP2); inositol triphosphate (IP_3_); diacylglycerol (DAG); protein kinase C (PKC); cyclic adenosine monophosphate (cAMP).

PAR-1 receptors are mainly activated by thrombin, but other mediators of the coagulation cascade can also cleave it. PAR-1 is ubiquitously found in endothelial cells, platelets, lungs, GI tract, immune cells, neurones and brain [11]. Following cleavage of PAR-1, there is activation of the G protein subunits G_12/13_, G_i_, and G_q_ (Figure 1A) [33,34,35,36]. Signalling via G_12/13_ leads stimulation of Rho guanine nucleotide exchange factor (RhoGEF), which in turn activates GTPase RhoA and is involved in cytoskeleton reorganization. Activation of G_i_ causes inhibition of adenylyl cyclase activity and hence a reduction in cyclic adenosine monophosphate (cAMP) production. Finally, G_q_ activation initiates the intracellular cascade starting with phospholipase-Cβ (CPLCβ) hydrolyzing phosphatidylinositol 4,5-bisphosphate (PIP2) to produce inositol triphosphate (IP_3_) and diacylglycerol (DAG). This leads to Ca^2+^ mobilization and an increase in the activity of protein kinase C (PKC) and other intracellular signalling enzymes. Different subunits of PKC facilitate the variety of downstream responses after PAR-1 activation [37,38,39,40].

A peculiar characteristic of PARs is that because its agonist is its own N-terminal sequence, there is no dissociation of the agonist from the receptor, so desensitization and termination mechanisms have to be tightly regulated. PAR-1 receptors are phosphorylated by G-protein coupled receptor kinases (GRKs) 3 or 5 and desensitized by β-arrestin binding, specifically β-arrestin 1, allowing the uncoupling of the G-proteins [12]. In contrast to other GPCRs, the β-arrestin associated with PAR-1 participates only in desensitization of the receptor, but not internalization [41]. Receptor internalization is primarily regulated by clathrin and dynamin activity. Adaptor protein complex 2 (AP-2) is essential for PAR-1 internalization, while lysosomal sorting and receptor recycling are mediated by the R4 subfamily of regulators of G protein signalling (RGS), viz. Rab11A and Rab11B [42,43,44,45]. Curiously, there is also an agonist-dependant response where activated protein C (APC) induces PAR-1 phosphorylation that does not result in effective internalization and the receptor accumulates in the cellular membrane [46,47].

### 2.2. PAR-1 and Joints

Within joints, PAR-1 has been located on fibroblasts, myoblasts, osteoblasts, chondrocytes, and synoviocytes of RA and OA patients [48,49,50]. In joint cartilaginous tissue, thrombin stimulates migration and proliferation of chondrocytes in a PAR-1-dependent manner [23]. Under normal circumstances, migration of chondrocytes can help with cartilage homeostasis and tissue healing; however, in joint diseases such as OA and RA, thrombin activation of those cells can initiate cartilage degradation [51], contributing to arthritis progression. Metalloproteinases, such as MMP13, are elevated in OA and RA joints and their catabolic properties lead to degradation of type II collagen through a PAR-1- and PAR-3-dependent pathway [52]. The same increase in expression of MMPs is observed with APC activity, but this seems to be through a mechanism other than PAR-1 activation [53].

In contrast, a protective role of PAR-1 in regulating osteoclast formation has also been reported. Kanno and collaborators showed that PAR-1 engagement can initiate a downstream response, culminating in phosphorylation of adenosine monophosphate kinase (AMPK) and inhibition of NF-kB signalling, resulting in reduced osteoclastogenesis. The authors showed an increase in urokinase plasminogen activating factor production following LPS treatment, and it is believed that subsequent plasmin production activated PAR-1 and reduced osteoclast formation [40]. These data were supported by another study showing that osteoclastogenesis was enhanced in PAR-1 knockout mice treated with TNF-alpha, indicating that PAR-1 acts as an inhibitor of osteoclast maturation in inflammatory joint diseases [54]. In a model of tibial damage, bone repair was found to be inhibited in PAR-1 knockout mice while thrombin improved bone marrow stromal cell proliferation in a PAR-1-dependent manner [24,55]. Thrombin is also able to inhibit osteoblast apoptosis, but in these cells this occurrence seems to be independent of PAR-1 activation [56].

In RA, synovial fibroblasts proliferate and form a hyperplastic and invasive tissue called pannus. Through PAR-1 cleavage, thrombin upregulates the expression of RANTES which then induces NF-kB and IL-6 expression, leading ultimately to synovial fibroblast differentiation and pannus formation [25,37,57,58]. In contrast, a protective role for PAR-1 in the joint has also been described. When treated with either thrombin, PAR-1-AP, or PAR-3-AP, synovial fibroblasts taken from OA patients have been shown to release heme-oxygenase-1 which is a protein with chondroprotective properties [38,59]. Thus, PAR-1 may be destructive or protective in the synovium depending upon whether the tissue is taken from a joint that has RA or OA, respectively.

The role of PAR-1 in joints has also been tested pre-clinically using animal models of arthritis. In the antigen-induced arthritis (AIA) model, it was observed that treatment with the PAR-1 antagonist hirudin led to a reduction in joint diameter, pannus formation, cartilage damage, fibrin deposition, and release of IL-1β [60]. Similar results were found using the collagen-induced arthritis (CIA) model, where PEG-hirudin decreased all the parameters cited above in addition to decreasing IL-12 expression and disease incidence and severity [61]. Repeating the AIA model in PAR-1-knockout mice revealed that arthritic animals had less cartilage and bone damage, diminished overall disease severity, reduced IL-1β, IL-6 and MMP13 levels in the synovium, and consequently less synovitis [62]. Again, it appears that PAR-1 contributes to the severity and pathophysiology of inflammatory joint disease.

In other studies, a link between PAR-1 and psoriatic arthritis has been observed. A transgenic mouse model that overexpresses kallikrein-related peptidase-6 (KLK6^+^) in keratinocytes, psoriatic arthritis markers such as IL-17A, IL-23 and IL-6 were all elevated [63]. Moreover, overexpression of KLK6^+^ caused increased expression of genes and proteins associated with psoriatic arthritis as well as pathological changes to the joint such as bone damage, synovitis, and impaired mobility. The PAR-1 antagonist vorapaxar inhibited the release of pro-inflammatory mediators from cultured keratinocytes, while PAR-1^−/−^ mice that overexpress KLK6^+^ did not show the same level of psoriatic arthritis [63]. These results indicate that sustained activation of PAR-1 by KLK6 plays an important role in the development of psoriatic arthritis.

With respect to pain, PAR-1 has divergent effects. The expression of PAR-1 in DRGs and its co-expression with TRPV_1_, SP and CGRP suggest a pro-nociceptive role for the receptor [64]. Thrombin injected into the dorsal horn of the spinal cord promoted the release of PGE_2_, leading to a decrease in mechanical withdrawal threshold [65], and an increase in glial cell expression [66]. Conversely, intraplantar injections of a synthetic PAR-1-AP was able to increase the withdrawal threshold in naïve animals without causing neuronal damage nor inflammation [67,68]. Following induction of inflammation with carrageenan, local injection of a PAR-1-AP was able to improve the hindpaw withdrawal threshold without affecting inflammation [67]. This analgesic effect of PAR-1 appears to involve the endogenous opioid system since naloxone was able to block PAR-1 responses [26]. The source of the opioids in inflamed tissues was found to be keratinocytes, as demonstrated by a rise in proenkephalin production by these cells.

On balance, PAR-1 appears to be predominantly protective in inflammatory joint disease but may be involved in degenerative activity in OA. Further studies are needed to affirm the role of PAR-1 in different joint conditions as well as determining its possible contribution to joint pain.

## 3. PAR-2

### 3.1. Receptor Pharmacology

PAR-2 is currently the most extensively characterized of all of the PARs. PAR-2 is the only PAR that does not have thrombin as the main endogenous activator. Rather, PAR-2 is predominantly cleaved by trypsin and tryptase, while other proteases such as neutrophil elastase and proteinase-3 are also known PAR-2 activators [14,15]. PAR-2 is highly expressed in epithelial cells of many systems such as lungs, liver, skin, blood vessels and the GI tract [69,70,71,72,73]. Expression of PAR-2 was also seen in mast cells [74], endothelial cells [75], different types of synoviocytes [76,77,78,79], joint sensory nerve fibres [29,30] and dorsal root ganglia [80], showing the importance of this receptor in many systems.

Similar to PAR-1, PAR-2 signalling involves G_q_, G_12/13_ and G_i_, although it is primarily mediated by G_q_ stimulating the PLCβ-IP_3_-DAG-PKC pathway (Figure 1B) [43,81]. In some cells, activation of PAR-2 induces arachidonic acid release and fast production of prostaglandins [73], suggesting the involvement of phospholipase A_2_ and cyclooxygenase-1. Other cells show the involvement of mitogen-activated protein kinase (MAPK) and ERK1/2 pathways after stimulation of PAR-2, as well as a non-G-protein-dependent pathway mediated by β-arrestin [43,82,83]. Biased signalling via the PLC-IP_3_-DAG-PKC pathway leads to Ca^2+^ mobilization from intracellular stores, while the MAPK pathway leads to signalling that is Ca^2+^-independent. The desensitization and termination of the PAR-2 signal is slightly different from PAR-1. Instead of using GRKs, PAR-2 phosphorylation is accomplished by PKC [81], which increases its affinity for clatharin, dynamin and β-arrestin 1 and 2, thereby facilitating the uncoupling of the receptor and its eventual internalization [84,85,86]. The internalization of PAR-2 involves β-arrestin with GTPases being important for endosome formation (Rab5a) and intracellular trafficking (Rab11a) [35,87].

### 3.2. PAR-2 and Joints

PAR-2 is widely expressed in multiple different tissues in the joint and is therefore likely to play a role in joint diseases such as OA and RA. In vitro studies showed that PAR-2 is expressed in osteoblasts and its activation induces calcium signalling and inhibition of osteoclast differentiation [76,88]. Compared to wild type animals, PAR-2^−/−^ mice have a lower expression of osteoblasts and osteoclasts, suggesting that PAR-2 is involved in the regulation of skeletal growth and bone repair [89]. Conversely, PAR-2 activation of osteoblasts from OA donors led to an increase in MMP1, MMP9, IL-6 and RANKL levels, indicating a bone resorptive effect in diseased joints [90].

Chondrocytes collected from OA patients show heightened levels of PAR-2 and exposure of these cells to inflammatory cytokines increased the expression of the receptor [77,79]. In contrast, TGF-β caused a downregulation of PAR-2 expression in OA chondrocytes but not in healthy cells [77,91], implying that various inflammatory mediators may differentially regulate the expression of PAR-2 in chondrocytes during joint disease. Matriptase is a serine protease that is rich in OA cartilage where it was shown to cause collagenolysis through PAR-2 activation [27]. Huang and colleagues (2019) used an antagonist of PAR-2 in both in vitro and in vivo models to elucidate the activity of PAR-2 in chondrocytes during OA as a possible treatment for the condition. They observed that AZ3451 inhibited PAR-2 expression and many of its downstream signalling pathways in vitro, reducing the expression of pro-inflammatory cytokines, collagen type II, and catabolic genes. In in vivo experiments, the antagonist inhibited cartilage destruction, decreased MMP13 levels and inhibited chondrocyte apoptosis [92]. Thus, PAR-2 may be a useful target to curtail cartilage damage during OA progression.

In synovial fibroblasts from human RA samples, the presence of PAR-2 receptors was detected, and stimulation of the receptor induced Ca^2+^ mobilization. Unlike chondrocytes, exposure of the synoviocytes to IL-1β and TNF-α did not increase the expression of PAR-2 in those cells [93]. In RA samples, mast cell tryptase activated PAR-2 on synovial fibroblasts inhibiting apoptosis via a rho kinase mechanism [94]. Both mast cell tryptase and a PAR-2-AP were able to induce synovial fibroblast proliferation in cells cultured from RA and OA patients with RA samples showing the greatest effect [95]. Analysis of venous blood samples from RA patients discovered that PAR-2 receptors were highly expressed on monocytes and T-cells, and generation of IL-6 by monocytes after PAR-2 activation was more prominent in blood from RA patients. PAR-2 expression in blood samples taken from OA patients revealed that the receptor levels were close to what was seen in control samples [96]. However, in OA patients with concomitant synovitis, PAR-2 receptor expression was elevated in the synovium and on macrophages, lymphocytes, and fibroblasts [97]. Measured levels of PAR-2 correlated with the severity of the synovitis, suggesting that PAR-2 is relevant in joint disease where there is ongoing inflammation. In other experiments, blocking PAR-2 reduced pannus invasion and proliferation, decreased the release of IL-17, IL-1β, and TNF-α, and ameliorated signs of OA via regulation of the MAPK/NF-kB pathway [50,98,99].

Direct injection of a PAR-2-AP into the joint was able to cause long lasting joint swelling, hyperemia, and synovial vasodilation in wild type mice, but not in their littermates lacking PAR-2 [100]. Degranulation of joint connective tissue mast cells with compound 48/80 produced an inflammatory response in wild type mice but not PAR-2 knockout animals, highlighting that mast cells are involved in the PAR-2 inflammation pathway [78]. Nociceptive behavior after an intraarticular injection of a PAR-2-AP was first observed by Helyes et al. (2010), where they were able to show that SLIGRL-NH_2_ could decrease paw withdrawal threshold and increase joint incapacitance [29]. This pro-algesic effect of PAR-2 activation was TRPV_1_-dependent since the selective TRPV_1_ antagonist SB366791 blocked this response. Articular injection of the more potent PAR-2-AP FLIGRL-NH_2_ induced spontaneous activity and increased firing of knee joint primary afferents in rats that was blocked by both TRPV_1_ and NK_1_ receptor antagonists [30]. In addition to a peripheral site of action for PAR-2, intra-thecal injection of FLIGRL-NH_2_ heightened mechanonociception in naïve rats [101]. Furthermore, spinal administration of the PAR-2 antagonist GB83 reduced secondary allodynia in models of chronic arthritis, but not in acute synovitis [101].

Cleaving serine proteases have also been shown to induce joint inflammation and pain in a PAR-2-dependent manner. For example, local administration of neutrophil elastase caused an increase in synovial blood perfusion, leukocyte trafficking, and nociceptive behaviour in wild type mice but not PAR-2^−/−^ [28]. Mast cell tryptase delivered into the knee caused synovial hyperaemia, oedema, and pain behaviour, which was reduced by chemical ablation of TRPV_1_ expressing neurones or in TRPV_1_^−/−^ animals [102]. Similarly, another PAR-2 cleaving enzyme, matriptase, can increase synovial perfusion in wild type mice but not PAR-2^−/−^ [27]. In addition to activating proteases, some enzymes can silence PAR activity by cleaving downstream of the canonical cleavage site. Calpain I, for example, disarms PAR-2 in joints, leading to a reduction in pain behaviour [103].

Experiments investigating a role for PAR-2 in chronic inflammatory joint disease found that in wild type mice, intra-articular injection of Freund’s complete adjuvant produced inflammatory cellular infiltration, synovial hyperplasia, and cartilage damage; however, none of these processes occurred in PAR-2^−/−^ mice, confirming that PAR-2 plays a role in joint inflammation [100]. PAR-2 has also been found to be involved in acute joint inflammation models where either deletion of the PAR-2 gene or inhibition of the receptor by RNA silencing reduced evidence of synovitis [100,104]. PAR-2^−/−^ mice were tested for their ability to develop RA in antigen-induced models and there was impaired development of the disease, indicating that PAR-2 is involved in the adaptative immune response to inflammatory joint disease [105,106].

PAR-2 has also been investigated in animal models of OA. Following joint destabilization, OA gradually developed in wild type but not PAR-2^−/−^ mice [107,108,109,110], indicating that PAR-2 is also involved in OA development. More recently, using chemical induction of OA, Muley and colleagues found that neutrophil elastase activity was increased in the joint of rats after induction of OA [111]. Pharmacological inhibition of this serine protease with alpha-antitrypsin or treatment with a PAR-2 antagonist blocked the initial inflammatory response to neutrophil elastase and reduced pain and peripheral neuropathy [111]. In a rodent model of chronic joint inflammation, alpha-antitrypsin reduced joint pain and leukocyte trafficking and exerted a chondroprotective effect [112]. It is clear that PAR-2 participates in the development of both RA and OA; therefore, inhibition of this receptor and its pathways presents a promising approach for the treatment of chronic joint diseases and their symptoms.

## 4. PAR-3

### 4.1. Receptor Pharmacology

The observation that platelets from PAR-1-knockout mice could still respond to thrombin led to the identification, cloning and characterization of PAR-3 [113,114]. A great limitation to the study of PAR-3 is the fact that synthetic activating peptides based on the receptor tethered ligand sequence do not activate PAR-3 [114]. It was later shown that these so-called PAR-3-Aps actually bound to both PAR-1 and PAR-2 instead [115]. As such, the signalling pathways of PAR-3 are still obscure, and it seems to play a more important role as a cofactor for other PARs as a regulator of their activity through heterodimerization [116,117,118].

### 4.2. PAR-3 and Joints

PAR-3 is co-expressed with PAR-1 on human chondrocytes but no functional activity of PAR-3 in those cells has been observed [23]. In synovial fibroblasts from RA samples, PAR-3 is co-expressed with PAR-1 and can cause a small Ca^2+^ influx itself, but not enough to alter cell activity [58]. In contrast, thrombin and a PAR-3-AP can stimulate production of heme oxygenase-1 in synovial fibroblasts from OA patients, and this effect can be inhibited by siRNA blockade of PAR-3 [38]. However, since the PAR-3-AP is not selective and PAR-3 is co-expressed with PAR-1 in synovial fibroblasts, it is feasible that these responses are actually a result of PAR-1 activation and PAR-3 is acting here as a cofactor to regulate PAR-1 activity. The main hindrance to PAR-3 biology has been the lack of selective PAR-3 agonists and antagonists. However, the recent discovery of a PAR-3 lipid tethered binding peptide (C660) may reveal a possible role for PAR-3 in arthritic conditions [119].

## 5. PAR-4

### 5.1. Receptor Pharmacology

PAR-4 was identified and cloned after the observation that platelets lacking PAR-1 and PAR-3 still responded to thrombin [120,121]. PAR-4 can be activated by thrombin, cathepsin G, tissue kallikreins, trypsin, and coagulation factors, via both G_q_ and G_12/13_ signalling pathways (Figure 1C). G_12/13_ follows the RhoGEF, GTPase RhoA, and downstream signalling pathways in order to alter the conformation of platelets and initiate aggregation. Engagement of the G_q_ second messenger system leads to the activation of PLCβ, production of IP_3_ and DAG, leading to Ca^2+^ mobilization and enhancement of PKC activity [122,123]. It has been shown that PAR-4 signalling and termination occurs at a much slower pace than PAR-1. First PAR-1 is activated and promotes an acute Ca^2+^ increase followed by a prolonged and sustained Ca^2+^ signal elicited by PAR-4 activation [124,125]. One of the reasons PAR-4 can sustain the signalling is based on the C-terminus being much shorter than other PARs and it having less sites for phosphorylation than are necessary for receptor desensitization and internalization [124]. Furthermore, PAR-4 internalization proceeds independently of β-arrestin but rather occurs via clatherin coating and endocytosis. Nevertheless, regulators of G protein signalling (RGS) eventually recognize the receptor and form complexes to terminate its signalling. Specifically, RGS2 interacts with G_q_ and RGS4 interacts with G_12/13_, inhibiting the downstream processes of these G proteins [126]. Upon internalization, PAR-4 is sorted between lysosomes for degradation or endosomes for recycling [122]. Interestingly, the recycling of PAR-4 is more efficient when it forms a dimer with PAR-2, resulting in a faster process of delivering PAR-4 to the cell membrane [127]. Dimerization with PAR-1 or PAR-3 can also influence PAR-4 activity by potentiating or modulating its signalling properties [128,129].

### 5.2. PAR-4 and Joints

PAR-4 receptors are expressed throughout the knee joint, as seen from positive immunohistochemical staining in articular cartilage, subchondral bone, menisci, synovium, mast cells, and chondrocytes. [31,32,130,131]. Intra-articular injection of the neuronal tracer fluorogold revealed that PAR-4 is expressed on joint primary afferents, suggesting that the receptor has the potential to modulate nociceptor firing. In a model of arthritis, the level of PAR-4 in the joint positively correlated with monocyte infiltration and synovial hypertrophy, indicating that PAR-4 plays a role in joint inflammation [97]. In contrast to the anti-inflammatory and analgesic effect of PAR-4 activation in skin and the gut [132], intraarticular injection of a PAR-4-AP caused a long-lasting increase in synovial blood flow, oedema formation, and an increase in mechanical and thermal pain [32]. Recently, it has been found that PAR-4 is involved in joint damage associated with articular bleeds. The accumulation of blood in a joint, for example, as a result of trauma, can lead to arthropathy, including synovitis and cartilage damage. Silencing of PAR-4 using siRNA reduced synovitis scores and hyaline cartilage destruction in mice with an intra-articular bleed [31]. It was suggested that this pro-arthritic effect was in part mediated by increased plasmin levels cleaving PAR-4 in the joint.

Electrophysiological recordings from joint primary afferents revealed an increase in nerve firing after close intra-arterial injection of a PAR-4-AP confirming a pro-nociceptive role for PAR-4 in joints. The inflammatory and nociceptive effects of PAR-4 activation could be blocked by a PAR-4 antagonist or the bradykinin B_2_ receptor antagonist HOE140 [130]. Conversely, the TRPV_1_ antagonist SB366791 did not influence PAR-4 responses. Pre-treatment of rats with a mast cell stabilizer also blocked mechanosensitization and pain implicating a mast cell-kinin mechanism for PAR-4 activity [131]. In other studies, silencing of PAR-4 with siRNA treatment reduced the severity of joint damage and plasmin activity in a model of blood-induced arthropathy [31]. Together, these data imply that inhibition of PAR-4 activity can be used to treat joint inflammation and pain.

## 6. Conclusions

**Figure 2:**
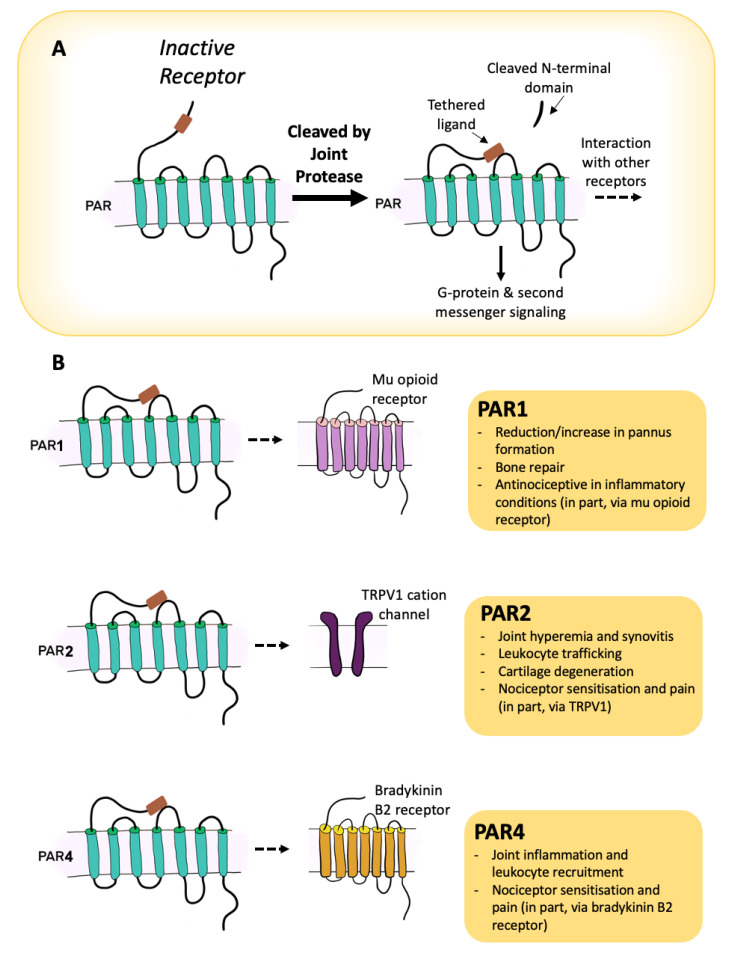
Overview of the role of PARs in the control of joint inflammation and pain. Activation of a PAR is achieved by cleavage of the receptor with a proteolytic enzyme to reveal a tethered ligand that binds to the second extracellular loop, leading to G protein coupling and signalling (**A**). In joints, PAR-2 and -4 tend to be pro-inflammatory and pro-nociceptive, whereas PAR-1 has some protective properties (**B**).

The family of PARs are richly expressed throughout joints and are associated with tissues associated with pain control, inflammation, and structural damage. On balance, PARs appear to be promoters of heightened inflammation and pain, while their contribution to joint destruction may be disease-dependent and requires further exploration (for summary, see Figure 2). Growing evidence seems to indicate that PAR-1 and PAR-2 can cause innate and neurogenic inflammation in different arthritis models, while PAR-4 seems to require platelet–leukocyte interactions, mast cell degranulation, and involvement of the kallikrein-kinin systems. When it comes to pain transmission, PAR-1 imparts an analgesic effect, whereas PAR-2 and PAR-4 support a pro-nociceptive outcome. The role of PAR-3 in joints is still unclear and we await better tools and techniques to unravel the function of this receptor in arthritis pathogenesis and symptom development. The dual effects of serine proteases to induce joint tissue catabolism and signal pain and inflammation make them promising candidates to redress the destructive and painful features of arthritis.

## Figures and Tables

**Table 1:** Known cleaving proteases and synthetic activating peptides for each of the PARs and their role in arthritis.

PAR	Activating Proteases	Synthetic Activating Peptides	Effect in Joints
PAR-1	Thrombin	SFLLRN-NH2	Chondroprotection [23]
	Granzyme A	TFLLRN-NH2	Bone repair [24]
	Plasmin		Pannus formation [25]
	Activating protein C		Anti-allodynic via an opioid mechanism [26]
	Trypsin		
	Factor Xa		
	Kallikrein- 4, 5, 6, 14		
	MMP-1		
	Cathepsin G		
	Proatherocytin		
	Pen C 13		
	Chymase		
PAR-2	Trypsin	SLIGRL-NH2	Cartilage degeneration [27]
	Mast cell tryptase	FLIGRL-NH2	Synovial hyperaemia and increased leukocyte trafficking [28]
	Factor Xa: Factor VIIa		TRPV_1_-dependent afferent sensitization and pain [29,30]
	Acrosin		
	Matriptase		
	Serine 11D		
	Trypsin		
	Granzyme A		
	Kallikrein-2, 4, 5, 6, 14		
PAR-3	Thrombin	N/A	N/A
PAR-4	Thrombin	AYPGKF-NH2	Joint damage [31]
	Trypsin	GYPGKF-NH2	Joint hyperaemia and oedema [32]
	Cathepsin G		Afferent sensitization and pain via mast cell degranulation and bradykinin activation [32]
	Trypsin IV		
	Mannin-binding SP-1		
	Plasmin		
	Factor Xa		
	Kallikrein-1, 14		
	C4a		

## Data Availability

Not applicable.
